# Immune Aging and Immunotherapy in Cancer

**DOI:** 10.3390/ijms22137016

**Published:** 2021-06-29

**Authors:** Melanie Kaiser, Maria Donatella Semeraro, Markus Herrmann, Gudrun Absenger, Armin Gerger, Wilfried Renner

**Affiliations:** 1 Molecular and Cell Biology Laboratory, The Salk Institute for Biological Studies, La Jolla, CA 92037, USA; 2 Clinical Institute of Medical and Chemical Laboratory Diagnostics, Medical University of Graz, 8036 Graz, Austria; maria.semeraro@medunigraz.at (M.D.S.); markus.herrmann@medunigraz.at (M.H.); wilfried.renner@medunigraz.at (W.R.); 3 Division of Oncology, Department of Internal Medicine, Medical University of Graz, 8036 Graz, Austria; gudrun.absenger@medunigraz.at (G.A.); armin.gerger@medunigraz.at (A.G.)

**Keywords:** T cells, aging, cancer, immune checkpoint inhibitors, biomarker, senescence, exhaustion

## Abstract

Immune functions decline as we age, while the incidence of cancer rises. The advent of immune checkpoint blockade (ICB) has not only revolutionized cancer therapy, but also spawned great interest in identifying predictive biomarkers, since only one third of patients show treatment response. The aging process extensively affects the adaptive immune system and thus T cells, which are the main target of ICB. In this review, we address age-related changes regarding the adaptive immune system with a focus on T cells and their implication on carcinogenesis and ICB. Differences between senescence, exhaustion, and anergy are defined and current knowledge, treatment strategies, and studies exploring T cell aging as a biomarker for ICB are discussed. Finally, novel approaches to improve immunotherapies and to identify biomarkers of response to ICB are presented and their potential is assessed in a comparative analysis.

## 1. Immunological Aging

### 1.1. Introduction

The immune system is a complex network of cells and proteins that does not only defend the body against foreign pathogens such as viruses or bacteria, but also clears out damaged or cancerous cells. It consists of two broad cellular responses. The first and instant line of defense is provided by the innate immune response, consisting of phagocytic cells and specific proteins that recognize conserved features of microorganisms called pathogen associated molecular patterns (PAMPs) through prototypically toll-like receptors (TLRs) [1]. The adaptive or acquired immune response on the other hand takes days to weeks to establish but offers superior selectivity for target antigens through specific surface receptors expressed on T and B lymphocytes. A hallmark of the adaptive immune response is that it gives rise to long-lived cells that can persist in a dormant state but can re-express effector functions rapidly after another encounter with their specific antigen. Despite major differences in their mechanism of action, synergism between innate and adaptive immunity is a prerequisite for an intact immune response [2]. However, as humans age, the immune system undergoes dramatic changes, culminating in losing the ability to protect against cancerous cells infections and foreign invaders. With increasing age, the innate immune functions continuously progress to create a characteristic pro-inflammatory environment, so called “inflammaging”, while the adaptive immune response declines, leading to massive T cell dysfunction [3,4,5]. This review article focuses on major changes regarding acquired immunity, especially on T cells, while remodeling of the innate immune system has comprehensively been reviewed elsewhere [6,7].

### 1.2. Immunosenescence, Senescence, Exhaustion, and Inflammaging

#### 1.2.1. Senescence

Cellular senescence is a permanent G1 cell cycle arrest that limits the lifespan of mammalian cells and prevents uncontrolled cell proliferation. This may be the consequence of oncogene activation, telomere erosion, and exposure to stressful stimuli such as reactive oxygen species, ionizing radiation, or chemicals. In the 1960s, Leonard Hayflick and Paul Moorehead reported their seminal finding that primary human cells cease to proliferate due to a finite number of cell doublings. This phenomenon turned out to be due to the erosion of telomeres, which induced a DNA damage response and was termed replicative senescence [8,9,10,11]. Arrested growth can also be triggered through oncogenic signaling, termed oncogene-induced senescence (OIS), resulting from an activating mutation of an oncogene, a tumor suppressor activation (p16^INK4a^) or loss (PTEN), epigenomic perturbations, and through adverse extrinsic stimuli such as DNA damage or oxidative stress, then called stress-induced premature senescence [12,13,14,15,16,17,18]. The master regulators for the initiation of a stable proliferative growth arrest are p53 and p16^INK4a^-pRb tumor suppressor pathways [19]. OIS is considered a potent defensive mechanism against tumors since it prevents proliferative growth of cancerous cells, whereas replicative senescence is mostly linked to the physiologic process of aging or the pathogenesis of various degenerative diseases [20]. Besides a permanent growth arrest, senescent cells show widespread changes in gene expression and chromatin organization [21]. In addition, these cells are metabolically and transcriptionally active. Unlike apoptosis, another cell-cycle exit program that results in cell death, senescent cells remain viable and most of them secrete numerous proinflammatory cytokines, chemokines, growth factors, and proteases, a feature termed senescence-associated secretory phenotype (SASP), which can have deleterious effects on the microenvironment [22,23]. While the SASP is expressed due to genomic, mitogenic, and oxidative perturbations (including oncogene activation), it is not present when the cyclin dependent kinases p16^INK4A^ or p21 are at play [24]. Although SASP is not yet fully understood, it has been proposed that it requires persistent DNA damage, which is not the case in ectopic expression of p16^INK4A^ or p21 [25]. The SASP is regulated by several pathways, including the ataxia telangiectasia mutated kinase (ATM), checkpoint kinase 2 (CHK2), p53, nuclear factor κB (NF-κB), CCAAT-enhancer-binding protein β (C/EBPβ), and p38 mitogen-activated protein kinase (MAPK) [26,27,28,29,30,31]. While the acquisition of SASP plays an important role, for example, in wound healing or embryogenesis, in the long term it can modulate neighboring cells including immune cells and can turn senescent fibroblasts into proinflammatory cells. These in return can promote tumor progression [23,30,32,33].

#### 1.2.2. Immunosenescence

Immunosenescence is a loosely defined term to conveniently designate weak immunity in the elderly [34]. It was first coined by Roy Walford who hypothesized that the normal process of aging in humans and animals is pathogenetically related to faulty immune processes [35]. The cardinal features of immunosenescence were later defined to be an impaired ability to respond to new antigens, a lingering level of low-grade inflammation, the accumulation of memory T cells, and a concurrent decrease of naïve T cells. Furthermore, it is characterized by a greater propensity for autoimmune responses and an unsustained memory response [36,37]. 

#### 1.2.3. Aging T Cells: Anergy, Exhaustion, and Senescence 

T cells are responsible for the establishment and maintenance of immune responses. They develop from hematopoietic stem cells in the bone marrow. Progenitor cells then migrate to and colonize the thymus where they develop into thymocytes, which undergo a series of maturation steps. Cells that do not meet certain selection criteria undergo apoptosis. Naïve T cells (CD4 and CD8 single positive cells) exit the thymus and circulate in the peripheral blood. CD4 T cells carry out various tasks, ranging from activation of cells of the innate immune system, B-lymphocytes, cytotoxic T cells, to playing a critical role in the suppression of the immune reaction [38]. T cells in the periphery consist of various subsets. These include naïve T cells, which can respond to new antigens, memory T cells, which are derived from previous antigen activations and maintain long-term immunity, and regulatory T cells (Tregs), which keep immune responses in check [39].

T cell generation during human adult life depends mainly on peripheral proliferation of naïve T cells [40]. For reasons yet to be elucidated, naïve CD8^+^ T cells are less successfully maintained with age than are naive CD4^+^ T cells [41]. Indeed, a lower number of circulating naïve CD8^+^ T cells is the most consistent and prominent marker of immune aging in healthy older adults [42]. At the end of their lifespan of 4-6 months, T cells can become senescent [43]. However, it is important to distinguish between senescence, anergy, and exhaustion, which have different origins but share similar characteristics. 

#### 1.2.4. Anergic T Cells

T cell anergy is a functionally defined state of hypo-responsiveness in which T cells neither proliferate nor produce the major T cell growth factor IL-2 following an antigen encounter and subsequent T cell receptor (TCR) ligation. Another characteristic in anergic CD4^+^ and CD8^+^ T cells is a defective production of inflammatory cytokines such as Interferon-gamma (IFN-γ) and tumor necrosis factor-alpha (TNF-α) [44,45,46]. Although there is no specific and characteristic surface marker determining anergic T cells, epigenetic factors such as histone modifications through Sirt1 or IKAROS as well as the transcription factor early growth response gene 2 (Egr2) have been described to help establish and maintain the anergic state [47,48,49,50].

#### 1.2.5. Exhausted T Cells

The term T cell exhaustion describes the progressive loss of effector function and reduced proliferative capacity triggered by chronic antigen stimulation either through chronic infection or tumor progression. In an exhausted state the T cell exhibits a decreased production of effector cytokines, such as IL-2, IFN-γ, or TNF [51], an increased chemokine expression, and persistently high levels of expression of multiple inhibitory receptors, such as programmed cell death protein 1 (PD-1) [52], T cell immunoglobulin and mucin domain-containing protein 3 (TIM-3) [53,54], lymphocyte-activation gene 3 (LAG-3) [55], cytotoxic T-lymphocyte-associated protein 4 (CTLA-4) [56], T cell immunoreceptor with Ig and ITIM domains (TIGIT) [57], CD160 [58], CD244 (2B4) [59], etc., leading to a repressed anti-tumor immune response, and a suppressive microenvironment [60,61]. However, Pawelec argues that CD8^+^ T cell exhaustion seems to be important to control and extend the immunological reserve required for maintaining surveillance of chronic infections [62]. The role of T cell exhaustion is therefore context-dependent with major importance for the outcome of autoimmune disease, infection, and cancer [63,64].

#### 1.2.6. Senescent T cells and T_emra_ Cells

**Figure 1:**
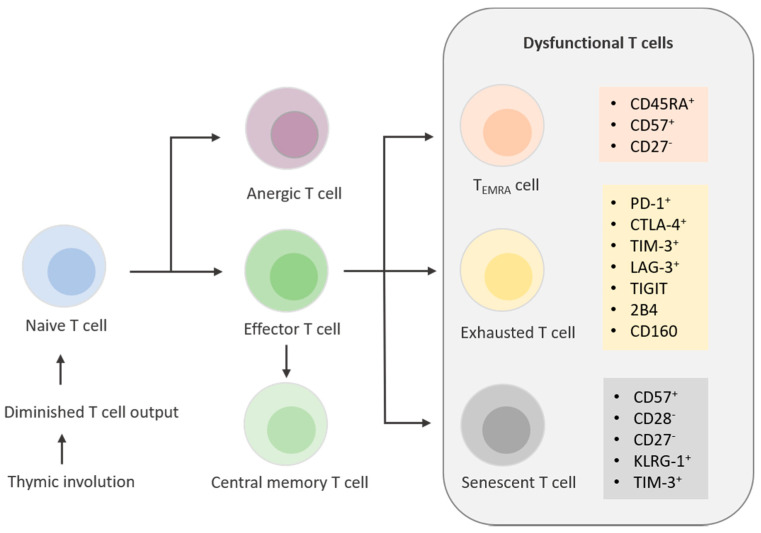
T cell differentiation and aging. Thymic involution leads to diminished T cell output in early adulthood. T cells further develop into anergic, effector, or central memory T cells. Over the course of immunological aging, T cells undergo drastic changes with different surface markers expressed on T_EMRA_ cells, exhausted T cells, and senescent T cells.

In contrast to exhaustion, which is controlled by extrinsic immunological regulatory mechanisms, senescence is controlled intrinsically by cellular stress responses [62]. T cell senescence is mainly regulated by MAPK signaling, whereas one of the main features of T cell exhaustion is inhibitory receptor-associated signaling [61]. A replicative senescent phenotype is further characterized by short telomeres together with a lack of co-stimulatory molecules CD28 and CD27 and elevated expression of senescence-associated-ß-galactosidase (SA-ß-Gal) [65]. These cells are found within the CD4^+^ as well as in the CD8^+^ compartment and feature an upregulation of CD57 and killer cell lectin-like receptor sub family G (KLRG1) surface markers (Figure 1). Studies have shown that CD57 is the most relevant marker for replicative senescence due to the severely impaired proliferative capacity of T cells expressing CD57 [66]. 

Another subset of CD27^−^CD28^−^ T cells that express CD45RA are termed effector memory T cells that re-express CD45RA (T_emra_ cells). These cells share several characteristics with senescent cells, such as loss of telomerase activity, an enzyme that can add new telomeric motifs to the end of telomeres. Furthermore, these cells exhibit decreased proliferative activity, increased levels of DNA damage, and, therefore, increased phosphorylated H2A histone family member X (γH2AX), which is part of the DNA damage response in senescent cells. Surprisingly, they do not have critically short telomeres, meaning in this subset of T cells, a different mechanism is leading to their senescent state, e.g., DNA damage due to reactive oxygen species (ROS) [67,68]. Furthermore, p16 and p21, which are involved in the regulation of the cell cycle, are upregulated due to the loss of CD27 and CD28 [69], ultimately inhibiting the transition from G1 to S phase of the cell cycle and leading to replicative senescence [70]. Senescent T cells also feature SASP, comprising suppressive cytokines such as IL-10 and TGF-ß, but also proinflammatory cytokines, such as TNF, IFNγ, IL-2, IL-6, and IL-8 [64,71,72]. The SASP in T cells is also governed by p38 MAPK signaling, contributing to age-associated inflammation [73]. 

#### 1.2.7. Inflammaging (Innate Immunity) Versus Immunosenescence (Adaptive Immunity)

Already in 1863, the father of modern pathology, Rudolph Virchov, hypothesized that there might be a connection between inflammation and cancer when he noted infiltrated immune cells in cancer lesions of inflamed tissue [74]. More recently, in 1986, Dvorak et al. argued that “tumors are healing wounds”. After decades of research, this has been confirmed and today it is an accepted fact that tumor cells generate a stroma for survival and growth by engaging the wound-healing system through overexpression of the vascular endothelial growth factor (VEGF) [75,76]. Indeed, chronic inflammation is not only involved in carcinogenesis, meaning the risk and onset of a malignant tumor, but also in its progression and metastatic diffusion thereafter. Furthermore, there is striking evidence that various other age-related diseases such as cardiovascular disease and type 2 diabetes amongst others, also comprise a systemic pro-inflammatory background, which is characterized with high levels of circulating interleukins such as IL-6, IL-1, TNF-α, and inflammatory mediators such as C-reactive protein (CRP) [77]. A seminal paper was published by Claudio Franceschi et al. in 2006 where the concept of aging was associated with a chronic, sterile, low-grade inflammation or “inflammaging”. Franceschi et al. argue that the main contributors to inflammaging are the following: dysfunctional mitochondria, defective autophagy/mitophagy (disposal of dysfunctional organelles), endoplasmic reticulum stress, activation of inflammasome by cell debris and misplaced self-molecules, defective ubiquitin/proteasome system (misfolded/oxidized proteins), activation of DNA damage response, age-related changes in the composition of gut microbiota (dysbiosis), and senescent T cells and their SASP [78]. Ostensibly, an increased production of inflammatory mediators contributes to an impaired adaptive immune response and, eventually, to immunosenescence. On the other hand, the decrease of the adaptive immune response reinforces the stimulation of the innate immune response. This occurs to protect organisms from infections in the event when acquired immunity fails, resulting in inflammaging. In fact, proinflammatory cytokines such as TNF-α, can induce CD28 downregulation, which is a hallmark of cellular senescence. To ensure an adequate immune response and sustained T cell proliferation, the co-engagement of CD28 with theTCR is key for the induction of IL-2 and its high affinity receptor CD25. It seems that inflammaging and immunosenescence progress in parallel in a downward spiral [79,80].

## 2. Aging, Cancer, and Immunotherapy

**Figure 2:**
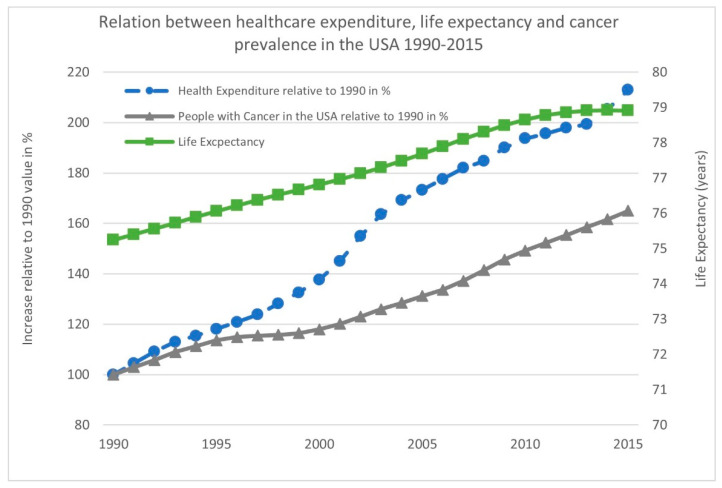
Relation between healthcare expenditure, life expectancy, and cancer prevalence in the USA from 1990 to 2015.

Aging is a major risk factor for cancer, one of the most significant causes of human morbidity and mortality. In both males and females, cancer is the number one cause of death in the age group of 60–79 years. With the proportion of people older than 60 growing faster than any other age group, this is placing an enormous economic and financial burden on society (Figure 2) [81,82,83].

Fortunately, immune checkpoint blockade (ICB) has revolutionized cancer treatment, providing unprecedented clinical benefits [84]. The significance of the discovery of immune checkpoints was underlined by the 2018 “Nobel Prize in Physiology or Medicine”, awarded to James Allison and Tasuku Honjo [85]. ICB removes inhibitory signals of T cell activation, which enables tumor-reactive T cells to overcome regulatory mechanisms and mount an effective antitumor response [86]. However, up to two-thirds of patients receiving immune checkpoint inhibitors (ICIs) in a palliative setting show therapy resistance, a fact that stresses the need to further investigate the mechanisms of treatment resistance and to design more effective therapeutic strategies. Another critical aspect of ICIs is that in rare cases they can cause life-threatening and/or even irreversible autoimmune toxicities. Additionally, rapid hyperprogressive disease (HPD) under these agents has been described, implying potentially deleterious effects of these pharmaceuticals [87]. Therefore, it is of great importance to improve patient selection and avoid toxicity as well as HPD in potential non-responders. 

So far, the focus of immune checkpoint therapy was on reversing the exhausted state of T cells via PD-1, programmed death-ligand 1 (PD-L1), and CTLA-4 inhibition. However, only a fraction of patients benefit from ICIs. Hence, there is considerable interest in the development of monoclonal antibodies (mAbs) targeting other immune checkpoints, e.g., LAG3. It has been shown that LAG3 and PD-1 are expressed particularly on TIL and act synergistically to promote tumor immune escape [88,89]. Therefore, a substantial amount of pre-clinical data has led to LAG3 being the third checkpoint to be targeted in the clinic with several therapeutics under investigation. [90,91].

PD-L1 expression and overall tumor mutational burden (TMB, the total number of mutations per coding area of a tumor genome) have been commonly used as clinical biomarkers. Nevertheless, both were hampered by being imperfect predictors of response [92]. PD-L1 expression is currently the most widely validated and accepted biomarker for the guidance of patient selection regarding anti-PD-1 or anti-PD-L1 therapy. However, the clinical utility of PD-L1 testing varies greatly between cancer types and treatment settings. There is a large variability in the types of PD-L1 assays, PD-L1 expression cutoffs, and types of cells tested for PD-L1 expression. It remains a significant challenge to decipher the various modes of testing and their application in routine clinical practice [93]. Although PD-L1 expression is not the optimal biomarker, it is to date the only relevant one in certain types of cancer, and it is clearly associated with better outcomes in non-small cell lung cancer (NSCLC), gastric and cervical cancer treated with PD-1, or bladder and triple negative breast cancer treated with PD-L1 [94]. However, this relationship is not as clear in combination regimens when chemotherapy or another ICI is added to the treatment. Furthermore, several studies provide evidence for efficacy of ICIs in patients with PD-L1 negative tumors [95]. Hence, the assessment of PD-L1 alone using IHC seems not to be an adequate determinant for patient selection in most cancers [96]. Therefore, the race is still on to find a predictive biomarker, especially a non-invasive one, but also to find therapeutic strategies beyond targeting PD-1/PD-L1 and CTLA-4 for an optimal and effective immunotherapeutic treatment of each patient.

### 2.1. Immune Aging of T Cells as a Biomarker for ICB and Evolving Treatment Regimens in Immunotherapy

Dysfunctional T cells can accumulate in the body simply by getting older, but also due to chronic infection or cancer. Malignant tumors can induce T cell exhaustion and senescence, which are two important dysfunctional states that coexist in cancer patients. These two states help sustain a suppressive tumor microenvironment by hindering effective antitumor immunity [72]. Several studies indicate that senescence occurs in human T cells in patients with chronic viral infections and in tumor-infiltrating lymphocytes (TILs) in various types of cancers, including ovarian, breast, lung, and colorectal cancer [97,98,99,100]. This indicates that T cell senescence is an alternative mechanism exploited by malignant tumors to evade the immune system [101]. This amongst other evidence cumulated in senescence being not only an emerging target for immunotherapy but also a peripheral predictive biomarker for immune checkpoint inhibitor therapy [72]. It is important to mention that changes in chromatin structure, especially through epigenetic alterations, are also associated with immune response and evasion of immune cells to ICB. Of note, the transition from a functional to dysfunctional state is sequential, with the progressive acquisition of chromatin remodeling and epigenetic reprogramming that eventually leads to a definitive state. Characterization of these transitional states and potential epigenetic predictive biomarkers have been extensively described and reviewed by Villanueva L et al., Xiao et al., and Ghoneim HE et al. [102,103,104].

#### 2.1.1. Biomarkers of the Periphery

When it comes to senescence in T cells, it needs to be considered that the enzyme telomerase reverse transcriptase (TERT) plays a pivotal role in maintaining telomere length and, therefore, the proliferative potential of human T cells, which is essential in mounting a proper immune response and eliminating cancer cells. As discussed in Section 1.2.1, when telomeres get too short, the cell enters a state called replicative senescence. The same mechanism applies to T cells, and for that reason, Weng et al. claimed that T cell function in the elderly may be improved by enhancing telomerase activity [105]. At least in adoptive cell transfer therapies, T cells with longer telomeres have been shown to be better candidates to persist and mediate anti-tumor effects due to their greater proliferative capacity [106,107]. In this context, the question arises whether short telomeres in T cells also play a role in ICI non-responders due to their limited proliferative potential and whether this could be used as a predictive biomarker along with already established biomarkers, such as TMB, and other very promising investigations described below. 

It was revealed that co-stimulation of the CD28 surface marker that is lost on senescent T cells is required for CD8^+^ T cell proliferation after PD-1 blockade [108,109]. This means that PD-1 limits T cell activity by inhibiting CD28 co-stimulation. Kamphorst et al. showed that CD28 signaling plays an important, if not major, role for responses to PD-1/PDL-1 blockade [110]. In addition, it was demonstrated that in lung cancer patients, PD-1^+^ CD8 T cells that proliferate in the peripheral blood after PD-1 blockade express CD28. These data hint at a selective proliferation of CD28^+^ cells through anti-PD-1 treatment, which implies further evaluation of CD28 for potential prediction of CD8^+^ T cell responses in cancer patients. In fact, Moreira et al. recently investigated whether senescence markers on T cells can predict response to ICB in melanoma patients [111]. They found that a loss of the surface markers CD27 and CD28 or the expression of Tim-3 and CD57 on peripheral T cells was associated with resistance to ICB, displayed in Table 1. Phenotyping T cells for senescence markers may thus help to predict ICI response. However, the cohort in that study consisted of only 10 patients. Larger studies are required to confirm those findings. 

Another study by Ferrara et al. concluded that circulating T cell senescence correlates with progression, HPD, and poor survival upon ICB in advanced NSCLC patients treated with single agent PD-1/PD-L1 inhibitors, as determined by assessing the percentage of a senescent immune phenotype (CD28^−^CD57^+^KLRG1^+^) in circulating CD8^+^ T cells [112]. Iwahori et al. reported that the cytotoxic activity of T cells at tumor sites is closely associated with that of T cells in the peripheral blood [113]. They claim that in NSCLC patients, the efficacy of the checkpoint inhibitor Nivolumab may be predicted by measuring the cytotoxicity of peripheral T cells in the blood. Furthermore, Yost et al. showed that following checkpoint inhibitor treatment, the expansion of tumor-infiltrating T cell clones did not arise from pre-existing TILs, but rather from novel clonotypes in the periphery. Interestingly, the T cell clones found in the TME before treatment were also there afterwards. Strikingly, the same exhausted T cell clones were found in the TME after therapy, meaning they did not acquire a non-exhausted state [114]. This implies that TILs have very limited potential to rejuvenate and that the expansion of TILs coming from the periphery plays a major role when it comes to ICB response. In fact, tracking of T cell clones using deep TCR sequencing after neoadjuvant PD-1 blockade in NSCLC patients showed that T cells that expanded in the periphery accumulated in the tumors of patients responding to therapy [115].

**Table 1:** Details of some factors of T cells that predict responses to immune checkpoint inhibitor therapy. MM (malignant melanoma), GC (gastric cancer), NSCLC (non-small lung cancer), burned-out CD8^+^ TIL (Ebo subset).

Type of Marker	Marker	Association with Response to Treatment	Cancer Type	References
**T cell biomarkers of the periphery**	CD27^−^	negative	MM	Moreira et al. [111]
	CD28^−^	negative	NSCLC, MM	Moreira et al. [111]; Ferrara et al. [112]
	CD-57^+^	negative	MM	Moreira et al. [111]
	TIM-3^+^	negative	MM	Moreira et al. [111]
	KLRG1^+^	negative	NSCLC	Ferrara et al. [112]
	Cytotoxic activity	positive	NSCLC	Iwahori et al. [113]
**T cell biomarkers of the TME**	TCF7	positive	MM	Sade-Feldman et al. [116]
	T cell inflamed GEP	positive	multiple cancer types	Christescu et al. [117]; Olson et al. [118]; Ayers et al. [119]
	Ebo subset	negative	NSCLC	Sanmamed [120]
	CXCL13	positive	multiple cancer types	Thommen et al. [121]; Litchfield et al. [122]
	CCR5	positive	multiple cancer types	Litchfield et al. [122]
	PD-1^+^	positive	MM, GC, NSCLC	Kumagai et al. [123]

#### 2.1.2. Biomarkers of the TME

T cells in the TME can also be used to predict immune responses to ICB in tumors. Sade-Feldman et al. found that the presence of a single transcription factor (*TCF7*) alone, visualized on CD8^+^ T cells of fixed tumor samples can predict clinical response to ICB in melanoma patients, suggesting that the state of T cells found in a patient’s tumor, in addition to the number of T cells and their spatial distribution, are also critical for induction of effective tumor immunity [117]. *TCF7* is part of the Wnt/b-catenin signaling pathway and plays a critical role in central memory formation, persistence, and self-renewal of CD8^+^ T cells [124,125]. 

Another recent study defined a new T cell subset of NSCLC patient tissue that expands within the TME, suggesting they are associated with primary resistance to anti-PD therapy. This burned-out CD8^+^ TIL subset (Ebo) is functionally distinct from previously described exhausted T cells. Ebo TIL do share features of exhausted T cells such as expression of co-inhibitory receptors (PD-1, LAG-3 TIM-3) and the loss of IFNγ production; however, they are highly proliferative. It seems that the mechanism of resistance to anti-PD therapy might be the excess of dysfunctional activated TILs where checkpoint inhibition prevents activated T cells from entering an apoptotic death program rather than restoring the function of exhausted T cells. These results indicate that depletion of Ebo cells may leave more space for the actual tumor fighting subset, such as effector CD8^+^ cells, or that measuring those subsets could serve as an additional biomarker of response to ICI therapy [121]. Of note, the response to ICB coming from TIL that are already present in the tumor and the response of tumor extrinsic T cells to therapy are not mutually exclusive and may represent an interdependent mechanism of response. In addition to the T cell–inflamed gene expression profile (GEP) [118,119,120], the expression of the immune cytokine CXCL13 is another emerging biomarker. The expression of CXCL13 seems to be not only predictive to ICB response when measured in baseline tumor tissue but is also associated with ICB outcome when evaluated in TIL exhibiting high expression of PD-1, in metastatic urothelial carcinoma and NSCLC, respectively [122,126]. The predictive value of CXCL13 was underlined by a recent study of Litchfield et al., who concluded that CXCL13 and CCR5 were T cell intrinsic markers for ICB response in a pan-cancer cohort [123]. Finally, Kumagai et al. recently showed that PD-1 expression balance between effector and regulatory T cells in the TME predicts the clinical efficacy of PD-1 blockade therapies and is superior to PD-L1 expression or TMB in patient cohorts with NSCLC, gastric cancer, or malignant melanoma [116]. This leads us to the conclusion that it is the combination of markers from the periphery with those in the TME that should lead to a more precise prediction of therapy response.

#### 2.1.3. Emerging Therapeutic Avenues

Senescence was long thought to be irreversible. However, in 2003, Beauséjour et al. showed that depending on expression of the pRB regulator p16, replicative senescence may not necessarily be permanent [127]. A relevant study by Lanna et al. found that senescent T cells use AMPK to recruit p38 to the scaffold TAB1. By blocking AMPK-TAB1-dependent p38 activation they were able to reverse the proliferative defect of senescent T cells [128]. Interestingly, inhibition of p38MAPK along with PD-1 inhibitors was shown in vitro to lead to restoration of the proliferative potential and TNF-α secretion in T_EMRA_ cells. This was not achievable by just blocking either pathway alone, demonstrating a relationship between T cell exhaustion and senescence [129]. Importantly, TNF-α is a cytokine that has the capability to suppress tumor cell proliferation and even to induce tumor regression [130,131]. Of note, p38 MAPK signaling is crucial for the induction of pro-inflammatory cytokines and plays a major role in controlling the SASP [26]. 

As mentioned before, the SASP of senescent cells can affect neighboring cells and can even induce senescence via paracrine signaling and change the tissue microenvironment. Therefore, it would be interesting to know whether altering SASP via the P38 MAPK pathway or using other interventions to reprogram (senostatic drugs) or eliminate (senolytic drugs) senescent cells in combination with immunotherapy would lead to an increased response to treatment. Lanna et al. suggest that targeting different molecules within the sestrin-MAPK activation complex may control senescence-related T cell functional changes. However, they also discuss that prolonged inhibition of sestrins could lead to proliferation of senescent cells that harbor DNA damage, potentially promoting malignancy [132], and conclude that short-term inhibition of sestrins might be a beneficial immunotherapeutic strategy [133]. Other studies targeting the MAPK pathway with BRAF and MEK inhibitors have shown divergent results. Enhanced antitumor immunity and tolerability have been shown in recent preclinical and clinical trials that combine anti–PD-1/PD-L1 with these inhibitors. In particular, MEK inhibition was associated with expansion of effector T cells and reduced exhaustion and apoptosis [134,135,136,137,138]. Gurusamy et al. recently demonstrated that p38 inhibition is not only crucial to reinvigorate senescent T cells but also important to block its activity in expanding less differentiated “younger” T cells to ensure their proliferative state [139], which is essential in adoptive T cell transfer-based (ACT) immunotherapy, e.g., chimeric antigen receptor (CAR) T cell therapy. Moreover, it has been suggested that combining CAR T cell therapy with PD-1 blockade could have beneficial effects in reversing T cell exhaustion [140], which makes it tempting to hypothesize that the combination of CAR T cell therapy, PD-1 inhibition, and blocking of p38 might be a beneficial cancer treatment regimen in some settings. In addition, it was demonstrated that senescence of T cells mediated by Tregs and tumors can be prevented or even reversed by TLR8 signaling [63,64,141]. Since the establishment of an immunosuppressive TME is a major barrier for successful tumor immunotherapy, targeting TLR8 signaling could be another strategy to enhance T cell function in addition to ICB. 

Several studies propose that cancer cells can also modify the metabolic program of T cells [142,143]. Liu et al. recently demonstrated that an unbalanced lipid metabolism is involved in T cell senescence in TIL in tumor mouse models and thereby strengthened the hypothesis that senescence in T cells is exploited by tumors to evade immune surveillance [144]. They propose that reprograming the lipid metabolism in T cells could enhance antitumor immunity. 

Emerging evidence also points at changes or modifications at the cellular level being crucial to gaining durable response in ICB. In this context the nuclear factor TOX seems to play a central role in priming T cells for exhaustion at the transcriptional and epigenetic levels [145,146,147,148,149,150,151]. This also drives the hypothesis that epigenetic modifiers might be useful in a combinatorial setting with ICB. Various other combinational treatment regimens targeting PD-1 and additional inhibitory receptors such as LAG-3 (discussed in chapter 2) or TIGIT already show promising results. More precisely, Chauvin et al. found that targeting TIGIT and PD-1 leads to synergistic reinvigoration of CD8 T cell function and proliferation in the periphery and tumor sites of melanoma patients [152]. 

## 3. Conclusion and Future Perspective

Over the past decade, efforts have been made to improve immunotherapies and to identify biomarkers of response to ICB. Despite all the progress in understanding and reversing T cell exhaustion and senescence signaling pathways, no ideal treatment regimen and no single marker has yet been found to perfectly discriminate between responders and non-responders. In the end, a multimodal therapeutic approach will be required to improve clinical outcomes. This implies several markers that need to be measured to find the best individualized treatment for each patient. 

Taken together, using a wide array of biomarkers of different origins in the tumor as well as in the periphery and combinational treatment to either eliminate or reprogram subsets of T cells will ultimately lead to more effective cancer treatment.
